# A *MYC*-rearrangement is a negative prognostic factor in stage II, but not in stage I diffuse large B-cell lymphoma

**DOI:** 10.1038/s41408-023-00971-y

**Published:** 2024-01-04

**Authors:** A. V. de Jonge, J. A. A. Bult, D. F. E. Karssing, M. Nijland, M. E. D. Chamuleau, M. Brink

**Affiliations:** 1 Department of Hematology, Amsterdam UMC Location Vrije Universiteit, Amsterdam, The Netherlands; 2 Cancer Center Amsterdam, Cancer Biology and Immunology, Amsterdam, The Netherlands; 3 Department of Hematology, University Medical Center Groningen, Groningen, The Netherlands; 4 Department of Research and Development, Netherlands Comprehensive Cancer Organization (IKNL), Utrecht, The Netherlands

**Keywords:** B-cell lymphoma, Cancer epidemiology, B-cell lymphoma

## Abstract

*MYC* oncogene rearrangements (*MYC*-R) negatively affect survival in patients with Ann Arbor stage III–IV diffuse large B-cell lymphoma (DLBCL), but their impact in limited stage (LS) I–II is unclear. Therefore, we assessed the impact of *MYC*-R on progression-free survival (PFS) and overall survival (OS) in LS DLBCL patients at the population level. We identified 1,434 LS DLBCL patients with known *MYC*-R status diagnosed between 2014 and 2020, who received R-CHOP(-like) regimens using the Netherlands Cancer Registry, with survival follow-up until February 2022. Stage I patients with (*n* = 83, 11%) and without (*n* = 650, 89%) a *MYC*-R had similar 2-years PFS (89% and 93%, *p* = 0.63) and OS (both 95%, *p* = 0.22). Conversely, stage II DLBCL patients with a *MYC*-R (*n* = 90, 13%) had inferior survival outcomes compared to stage II patients without a *MYC*-R (*n* = 611, 87%) (PFS 70% vs. 89%, *p* = 0.001; OS 79% vs. 94%, *p* < 0.0001). Both single *MYC*-R (single hit, *n* = 36) and concurrent *BCL2* and/or *BCL6* rearrangements (double/triple hit, *n* = 39) were associated with increased mortality and relapse risk. In conclusion, in stage II DLBCL a *MYC*-R is negatively associated with survival. In stage I DLBCL, however, survival outcomes are excellent irrespective of *MYC*-R status. This challenges the diagnostic assessment of *MYC*-R in stage I DLBCL patients.

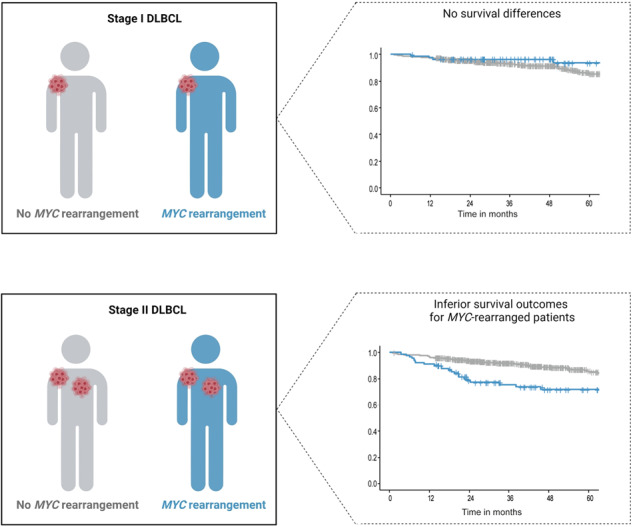

## Introduction

Diffuse large B-cell lymphoma (DLBCL) is the most common subtype of non-Hodgkin lymphoma and is generally treated with immunochemotherapy R-CHOP (rituximab, cyclofosphamide, doxorubicin, vincristine, and prednisone) [1–3]. However, the clinical outcomes of DLBCL patients are heterogeneous, which can at least partially be attributed to the variety in the genetic landscape in DLBCL patients [4–6]. A translocation of the *MYC* oncogene, detected with fluorescent in situ hybridization (FISH) in approximately 10–15% of all DLBCL cases, is one of the genetic aberrations associated with inferior prognosis [7].

The 5-year overall survival (OS) in patients with a *MYC* rearrangement (*MYC*-R) ranges from 35–55% as compared to 72% in patients without a *MYC*-R [8, 9]. Five-year progression-free survival (PFS) is lower in patients with a *MYC*-R (31–55%) compared to patients without a MYC-R (66%) [8, 9]. However, these associations are mainly observed in patients with advanced-stage DLBCL (Ann Arbor stage III–IV) [8, 9].

Impaired survival is most prominent in patients with a *MYC*-R combined with a rearrangement of the *BCL2* and/or *BCL6* gene (so-called ‘double hit’ [DH] and/or ‘triple hit’ [TH] high-grade B-cell lymphoma (DH/TH HGBL), especially when the fusion partner of *MYC* is the *IgH* locus [9]. To improve survival outcomes, advanced-stage DH/TH HGBL patients are usually treated with more intensive immunochemotherapeutic regimens, such as DA-EPOCH-R (dose-adjusted etoposide, prednisone, vincristine, cyclophosphamide, doxorubicin, and rituximab), or R-CHOP plus lenalidomide, although randomized controlled trials supporting this are lacking [10, 11]. Notably, in the aforementioned studies, stage II patients were grouped together with stage III and IV. As a result, the distinct prognosis for stage II patients remains unexplored. Besides, it remains unclear whether differences in clinical course between limited (stage I and II) and advanced-stage DLBCL should be attributed to early disease detection or to distinct biologic features, including the prognostic impact of a *MYC*-R [12].

Two previous studies where limited-stage (LS) DLBCL was defined as stage I or stage II, showed that the complete remission (CR) rate in DH patients was lower, but survival rates were similar as compared to patients without a DH (2-year PFS 74% and 78%, and 2-year OS 81% and 86%, respectively) [13, 14]. A third study reported an inferior relapse-free survival in DH/TH compared to non-DH/TH DLBCL [15]. However, due to the limited number of patients with a *MYC*-R without any distinction between stage I and stage II DLBCL, the impact of *MYC*-R on survival in stage I and stage II separately remains uncertain. Therefore, the aim of this study was to assess the impact of an *MYC*-R on survival outcomes for stage I and stage II DLBCL patients in the Netherlands.

## Methods

### Registry and study population

The nationwide population-based Netherlands Cancer Registry (NCR) is maintained and hosted by the Netherlands Comprehensive Cancer Organization (IKNL) and has nationwide coverage of at least 95% of all malignancies since 1989 [16]. The NCR relies on comprehensive case notification through the Nationwide Histopathology and Cytopathology Data Network and the Nationwide Registry of Hospital Discharges (i.e., inpatient and outpatient discharges). Information on topography and morphology, hospital, type of diagnosis, the World Health Organization (WHO) performance score, LDH level, and presence of *MYC*, *BCL2*, and/or *BCL6* rearrangements, and first-line therapy is routinely recorded by trained registrars of the NCR through retrospective medical records review. Information on the last known vital status for all patients (i.e., alive, dead, or emigration) is obtained through annual linkage with the Nationwide Population Registries Network that holds vital statistics on all residents of the Netherlands.

**Fig. 1:**
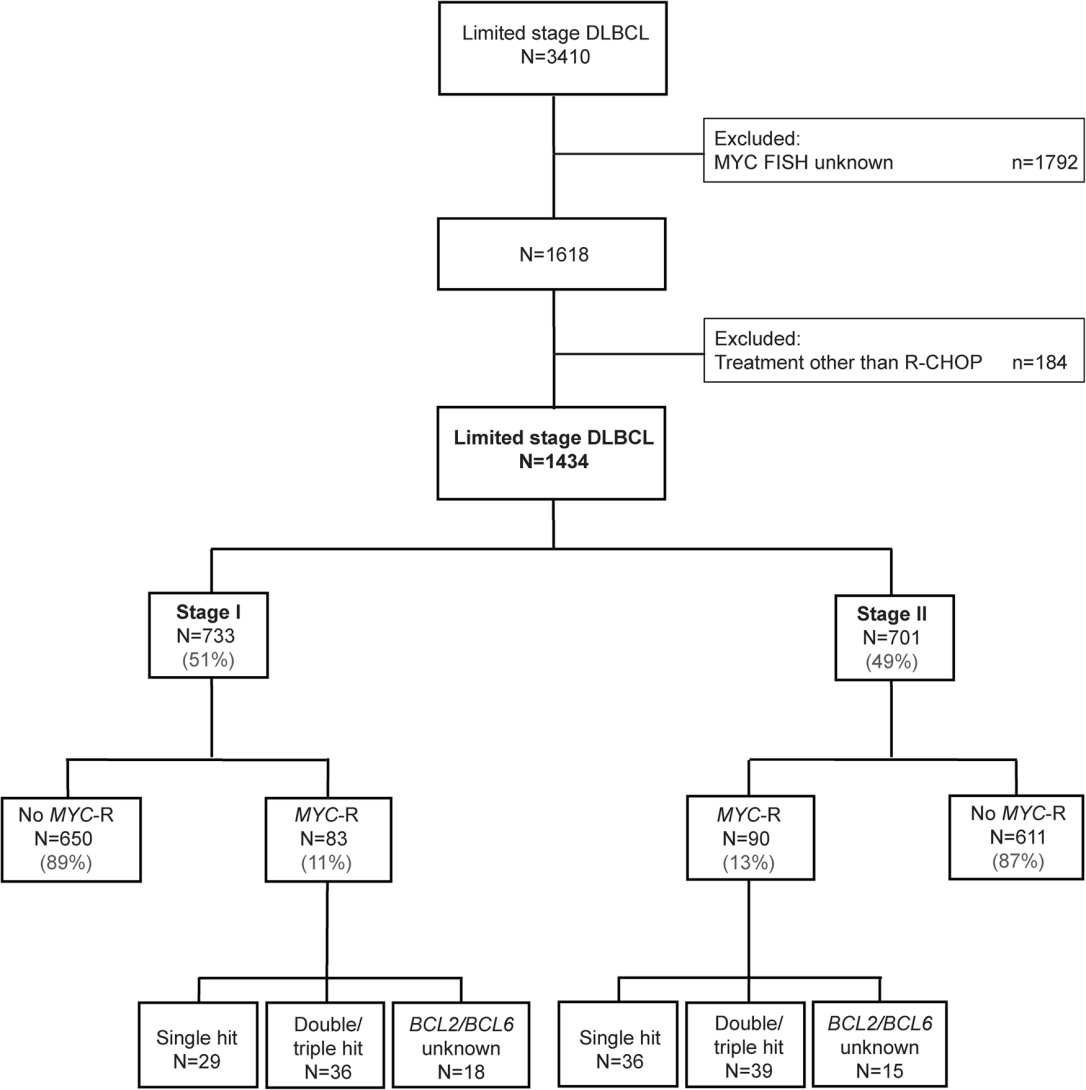
Flow chart of patient selection. Flow chart of limited stage I (*n* = 733) and stage II (*n* = 701) patients included in the analyses. *MYC*-R; *MYC* gene rearrangement, single hit; *MYC*-R only, double/triple hit; *MYC*-R with concurrent *BCL2* and/or *BCL6* rearrangements.

Patients aged ≥18 years with de novo stage I(E) or stage II DLBCL diagnosed between January 1st, 2014 and December 31st, 2020 were identified in the Netherlands Cancer Registry (NCR), using the International Coding System of Disease—Oncology (ICD-O) of the WHO, morphology code 9680/3. Stages I and II were defined according to the Ann Arbor staging system, determined by physician assessment. The increased use of FDG-PET and CT for the staging of aggressive lymphoma helps to more accurately distinguish those patients who truly have stage I from those who have stage II disease from 2014 onwards. However, it was not until 2018 that FDG-PET and CT were implemented as diagnostic tools at the national level. Patients with an unknown *MYC*-R status (*n* = 1792, 53%), and patients who received treatment other than R-CHOP-like regimens (*n* = 184, Supplementary Table 1) were excluded, leaving 1434 patients for all analyses (Fig. 1).

In the current study, patients with only a *MYC*-R were categorized as single hit (SH) B-cell lymphoma, whereas patients with a *BCL2* or *BCL6* rearrangements in addition to *MYC*-R were defined as double hit (DH) HGBL and patients with *MYC*, *BCL2* and *BCL6* rearrangements as triple hit (TH) HGBL according to the revised 4th edition of the WHO classification (2016) [17].

Patients were divided into 4 R-CHOP(-like) treatment groups: 1) 6–8 cycles R-CHOP, 2) abbreviated (3 cycles) R-CHOP plus radiotherapy (RT), 3) less intensive R-CHOP-like regimens, such as R-miniCHOP, rituximab combined with etoposide instead of doxorubicine (R-CEOP) or without etoposide or doxorubicin (R-CVP), and 4) more intensive R-CHOP-like regimens, such as rituximab combined with cyclophosphamide, doxorubicin, vincristine, etoposide, and prednisolone (R-CHOEP), and dose-adjusted etoposide, prednisone, vincristine, cyclophosphamide, doxorubicin, and rituximab (DA-EPOCH-R).

According to the Central Committee on Research Involving Human Subjects in the Netherlands (CCMO), this type of observational study does not require the approval of an ethics committee. The use of anonymized data for this study has been approved by the NCR Privacy Review Board.

### Endpoints

The primary endpoint of this study was overall survival (OS) defined as the time between the date of diagnosis and death by any cause. Secondary endpoints were progression-free survival (PFS) and best response. PFS was defined as the time between initial diagnosis and relapse or death by any cause. The best response, i.e., complete remission (CR), or stable/progressive disease (SD/PD) to first-line treatment, was determined through physician assessment and routinely collected by trained registrars of the NCR through retrospective medical record review.

### Statistical analysis

Analyses were performed separately for patients with stage I and stage II disease, as stage I patients more commonly receive abbreviated chemotherapy with RT. Comparisons between patients with and without a *MYC*-R were made using the Pearson *χ*^2^ test and the Kruskall–Wallis test for categorical and continuous variables, respectively. Kaplan–Meier estimates were used to analyze OS and PFS between patients with and without a *MYC*-R. Survival differences between patients with and without a *MYC*-R were performed using a log-rank test. Survival follow-up was cut off on February 1^st^, 2022. Patients diagnosed between 2014 and 2018 were actively followed for the occurrence of relapse, while patients diagnosed in 2019 or 2020 were not. As a consequence, only relapses within 1 year post-diagnosis were identified for patients diagnosed in 2019 or 2020. Therefore, patients diagnosed in 2019 or 2020 and alive without relapse were censored at 1 year of follow-up.

The impact of MYC status on risk of mortality and relapse was evaluated by using a multivariable Cox proportional hazard regression analysis, thereby evaluating age per year increment, sex, serum LDH, WHO performance score, number of extranodal sites, and treatment received as covariables. The results from the Cox regression analyses produced hazard ratios (HRs) with associated 95% confidence intervals (Cl). The proportional hazard assumption was tested based on the Schoenfeld residuals. All variables were introduced in the multivariable regression model simultaneously, thereby using a backward selection method to exclude stepwise covariables with a *p*-value below 0.05.

To investigate the potential benefit of intensive chemotherapy regimens in *MYC*-R patients, we performed a sensitivity analysis including stage II patients who were treated with R-CHOP (*n* = 57) or treated with more intensive chemotherapy regimens (*n* = 22).

*p*-values < 0.05 were considered statistically significant. All statistical analyses were performed in R versions 4.0.3 and 4.2.2.

## Results

In total, 1,434 LS DLBCL patients with known *MYC*-R status, diagnosed between 2014 and 2020, and treated with R-CHOP(-like) regimens, were identified in the NCR including 733 (51%) stage I patients and 701 (49%) stage II patients (Fig. 1).

### Clinical characteristics of stage I DLBCL patients

In stage I patients, 83 (11%) had a *MYC*-R of whom 36 (43%) were DH/TH HGBL and 18 (22%) had unknown BCL2 and BCL6 status (Fig. 1 and Table 1). *MYC*-R patients were more often male (*p* < 0.01), but other baseline characteristics did not significantly differ between patients with and without a *MYC*-R (Table 1).

**Table 1:** Baseline characteristics of stage I DLBCL patients by *MYC*-R status.

	No *MYC*-R	*MYC*-R	*p*-value
	(*N* = 650)	(*N* = 83)	
Age (years)			
Mean (SD)	64.9 (13.4)	63.2 (15.2)	0.37
Median [min, max]	68.0 [21.0, 89.0]	66.0 [18.0, 89.0]	
Sex			
Male	382 (58.8%)	62 (74.7%)	0.0074
Female	268 (41.2%)	21 (25.3%)	
WHO performance score			
0	288 (44.3%)	44 (53.0%)	0.44
1	113 (17.4%)	12 (14.5%)	
≥2	31 (4.8%)	2 (2.4%)	
Unknown	218 (33.5%)	25 (30.1%)	
LDH level			
Within reference range	502 (77.2%)	67 (80.7%)	0.6
Elevated	129 (19.8%)	15 (18.1%)	
Unknown	19 (2.9%)	1 (1.2%)	
Extranodal localizations (no.)			
None	308 (47.4%)	38 (45.8%)	0.87
1	340 (52.37%)	45 (54.2%)	
Unknown	2 (0.3%)	0 (0%)	
IPI-score			
Low	400 (61.5%)	55 (66.3%)	0.55
Low-intermediate	66 (10.2%)	9 (10.8%)	
High-intermediate	11 (1.7%)	0 (0%)	
Unknown	173 (26.6%)	19 (22.9%)	
Rearrangement			
No *MYC*-R	650 (100%)	0 (0%)	NA
Single hit	0 (0%)	29 (34.9%)	
Double/triple hit	0 (0%)	36 (43.4%)	
Missing *BCL2/BCL6*	0 (0%)	18 (21.7%)	
Duration of follow-up (months)			
Mean (SD)	43.8 (23.0)	46.7 (22.9)	0.24
Median [Min, Max]	41.5 [1.18, 96.9]	47.1 [5.75, 96.6]	

Out of the 83 *MYC*-R stage I patients, 40 patients received 3 cycles of R-CHOP plus RT (49%), and 35 received 6–8 cycles of R-CHOP (42%, Supplementary Fig. 1A and Supplementary Table 2). Three patients received less intensive chemotherapy (4%) and five patients received more intensive chemotherapy regimens (6%). Among the 650 patients without an *MYC*-R, similar treatment distributions were observed: 326 received 3 cycles of R-CHOP plus RT (50%), 275 patients received 6–8 cycles of R-CHOP (42%), and the remaining patients received less intensive chemotherapy (48,7%), or a more intensive chemotherapy regimen (*n* = 1; 0.2%).

### Outcome of stage I DLBCL patients

For stage I patients, CR rates were both 89% for patients with and without a *MYC*-R (*p* = 0.58).

**Fig. 2:**
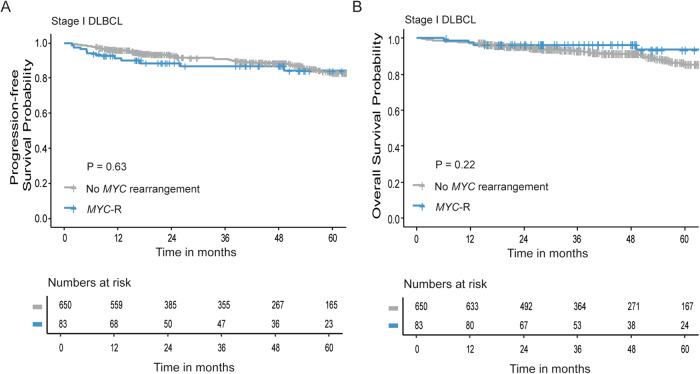
Survival analysis in stage I DLBCL patients stratified for *MYC* rearrangement status. **A**, **B** Progression-free survival (**A**) and overall survival (**B**) analyses in stage I DLBCL patients with (blue, *n* = 83) or without (gray, *n* = 650) a *MYC* rearrangement (*MYC*-R), using Kaplan–Meier survival analysis.

Median follow-up was similar between patients with (41 months) and without a *MYC*-R (47 months, *p* = 0.24). The 2-year PFS in patients with stage I disease with and without a *MYC*-R was similar (89% (95% CI 82–96%) and 93% (95% CI 91–95%), respectively, *p* = 0.63; Fig. 2A). Two-year OS was 95% (95% CI 93–97%) for *MYC*-R patients as well as for patients without a *MYC*-R (95% CI 92-100%, *p* = 0.22; Fig. 2B).

In a multivariable analysis, where we assessed the impact of *MYC* and *BCL2* and/or *BCL6* rearrangements on the risk of mortality and relapse, SH and DH/TH were not associated with risk of mortality (Supplementary Table 3) or risk of relapse (Supplementary Table 4) compared to patients without a *MYC*-R.

Older age, elevated LDH, WHO performance score 2–4, and male gender were associated with a higher mortality risk (Supplementary Table 3) and relapse risk (Supplementary Table 4). Treatment with three cycles of R-CHOP plus RT was associated with a lower relapse risk as compared to 6–8 cycles of R-CHOP (Supplementary Table 4).

### Clinical characteristics of stage II DLBCL patients

In stage II patients, 90 (13%) had a *MYC*-R of whom 39 (43%) were DH/TH HGBL and 15 (17%) had unknown BCL2 and BCL6 status (Fig. 1 and Table 2). Baseline characteristics did not differ between patients with and without *MYC*-R patients (Table 2).

**Table 2:** Baseline characteristics of stage II DLBCL patients by *MYC*-R status.

	No *MYC*-R	*MYC*-R	*p*-value
	(*N* = 611)	(*N* = 90)	
Age (years)			
Mean (SD)	63.6 (14.4)	63.4 (15.3)	0.92
Median [Min, Max]	67.0 [19.0, 89.0]	66.5 [18.0, 95.0]	
Sex			
Male	347 (56.8%)	53 (58.9%)	0.79
Female	264 (43.2%)	37 (41.1%)	
WHO performance score			
0	245 (40.1%)	40 (44.4%)	0.81
1	119 (19.5%)	17 (18.9%)	
≥2	22 (3.6%)	4 (4.4%)	
Unknown	225 (36.8%)	29 (32.2%)	
LDH level			
Within reference range	387 (63.3%)	50 (55.6%)	0.22
Elevated	219 (35.8%)	40 (44.4%)	
Unknown	5 (0.8%)	0 (0%)	
Extranodal localizations (no.)			
None	357 (58.4%)	55 (61.1%)	0.56
1	247 (40.4%)	35 (38.9%)	
2 or more	7 (1.1%)	0 (0%)	
IPI-score			
Low	340 (55.6%)	44 (48.9%)	0.45
Low-intermediate	91 (14.9%)	19 (21.1%)	
High-intermediate	12 (2.0%)	2 (2.2%)	
Unknown	168 (27.5%)	25 (27.8%)	
Rearrangement			
No *MYC*-R	611 (100%)	0 (0%)	NA
Single hit	0 (0%)	36 (40.0%)	
Double/triple hit	0 (0%)	39 (43.3%)	
Missing *BCL2/BCL6*	0 (0%)	15 (16.7%)	
Duration of follow-up (months)			
Mean (SD)	42.9 (21.5)	40.1 (24.9)	0.12
Median [min, max]	41.7 [0.526, 96.1]	32.8 [3.42, 93.3]	

Out of the 90 *MYC*-R stage I patients, 57 patients received 6–8 cycles of R-CHOP (63%). The remaining *MYC-*R patients received 3 cycles of R-CHOP plus RT (*n* = 3; 3%), less intensive chemotherapy (*n* = 8; 9%), or more intensive chemotherapy regimens (*n* = 22; 24%, Supplementary Fig. 1B and Supplementary Table 2). Among the 611 patients without a *MYC*-R, similar treatment distributions were observed, with 543 patients (89%) receiving 6–8 cycles of R-CHOP, and the remaining patients R-CHOP plus RT (*n* = 22; 3%), less intensive chemotherapy (*n* = 43; 7%) or more intensive chemotherapy regimens (*n* = 3; 1%).

### Outcome of stage II DLBCL patients

CR rate was lower in stage II patients with a *MYC*-R compared to patients without a *MYC*-R (81% vs. 89%, respectively; *p* = 0.02). Median follow-up was similar between patients with (42 months) and without (33 months, *p* = 0.12) a *MYC*-R.

**Fig. 3:**
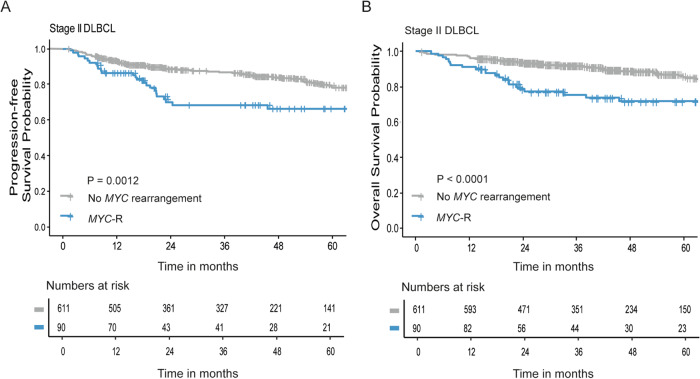
Survival analysis in stage II DLBCL patients stratified for *MYC* rearrangement status. **A**, **B** Progression-free survival (**A**) and overall survival (**B**) analyses in stage II DLBCL patients with (blue, *n* = 90) or without (gray, *n* = 611) a *MYC* rearrangement (*MYC*-R), using Kaplan–Meier survival analysis.

The 2-year PFS was lower in *MYC*-R patients compared to patients without a *MYC*-R (70% (95% CI 60–81%) and 89% (95% CI 86–91%), respectively, *p* = 0.0012; Fig. 3A).

The 2-year OS was also lower in *MYC*-R patients compared to patients without a *MYC*-R (79% (95% CI 70–88%) vs. 94% (95% CI 92–96%), respectively, *p* < 0.0001; Fig. 3B).

Within *MYC*-R patients, SH and DH/TH patients had a comparable 2-year PFS (60% (95% CI 43–84%) and 70% (95% CI 56% and 87%), respectively, *p* = 0.83, Supplementary Fig. 2A) and OS (78% (95% CI 65–94%) and 73% (95% CI 60–89%), *p* = 0.57, Supplementary Fig. 2B).

In a multivariable analysis, SH and DH/TH were associated with a higher risk of mortality (HR 2.27, 95% CI 1.12–4.59, *p* = 0.02, and HR 3.55, 95% CI 1.90–6.65, *p* < 0.01, respectively, Supplementary Table 5) and risk of relapse (HR 2.20, 95% CI 1.17–4.14, *p* = 0.01, and HR 2.08, 95% CI 1.14–3.79, *p* = 0.02, respectively, Supplementary Table 6) compared to patients without a *MYC*-R.

Furthermore, involvement of ≥1 extranodal site was associated with a higher mortality risk and older age was associated with a higher mortality and relapse risk (Supplementary Tables 5 and 6).

### Outcome of stage II DLBCL MYC-R patients treated with more intensive chemotherapy

**Fig. 4:**
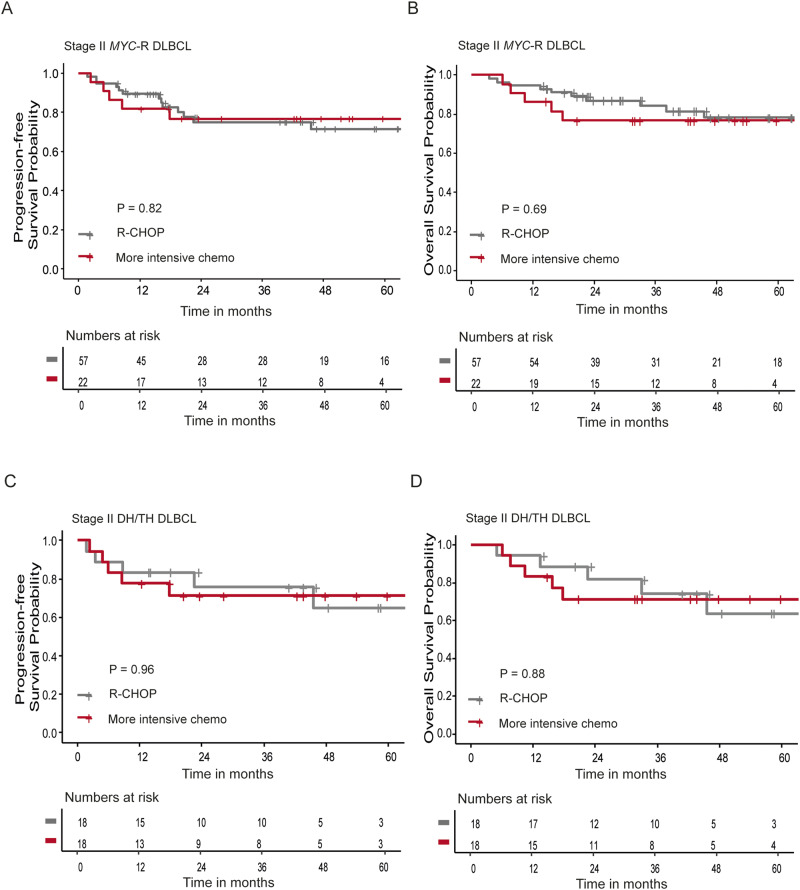
Survival analysis in stage II *MYC*-R DLBCL patients stratified for treatment. **A**, **B** Progression-free survival (**A**) and overall survival (**B**) analyses in stage II *MYC-*R DLBCL patients including single hit and double/triple hit lymphoma treated with R-CHOP (gray, *n* = 57) or more intensive chemotherapy regimens (red, *n* = 22), using Kaplan–Meier survival analysis. **C**, **D** Progression-free survival (**C**) and overall survival (**D**) analyses in stage II DLBCL double hit patients treated with R-CHOP (gray, *n* = 18) or more intensive chemotherapy regimens (red, *n* = 18), using Kaplan–Meier survival analysis.

Given the inferior outcomes of stage II *MYC*-R DLBCL patients, we aimed to investigate the potential benefit of intensive chemotherapy regimens in *MYC*-R patients in a sensitivity analysis. Survival outcomes in *MYC*-R patients treated with more intensive treatment regimens (*n* = 22) were compared to patients treated with 6–8 cycles of R-CHOP (*n* = 57). Median follow-up time was similar between the two groups (Table 3). The 2-year PFS and 2-year OS did not differ between the two treatment groups (*p* = 0.82, Fig. 4A, and *p* = 0.69, Fig. 4B, respectively). In DH/TH patients (*n* = 18 in both treatment groups, Table 4), the 2-year PFS and 2-year OS did not differ between the two treatment groups either (*p* = 0.96 Fig. 4C, and *p* = 0.88, Fig. 4D, respectively).

**Table 3:** Baseline characteristics of stage II *MYC*-R DLBCL patients by treatment.

	R-CHOP	More intensive chemotherapy	*p*-value
	(*N* = 57)	(*N* = 22)	
Age (years)			
Mean (SD)	62.2 (15.8)	61.3 (14.1)	0.7
Median [min, max]	65.0 [18.0, 94.0]	66.0 [30.0, 82.0]	
Sex			
Male	37 (64.9%)	13 (59.1%)	0.83
Female	20 (35.1%)	9 (40.9%)	
LDH level			
Within reference range	34 (59.6%)	8 (36.4%)	0.11
Elevated	23 (40.4%)	14 (63.6%)	
Extranodal localizations (no.)			
None	35 (61.4%)	12 (54.5%)	0.76
1	22 (38.6%)	10 (45.5%)	
2 or more	0 (0%)	0 (0%)	
IPI-score			
Low	31 (54.4%)	8 (36.4%)	0.044
Low-intermediate	6 (10.5%)	8 (36.4%)	
High-intermediate	1 (1.8%)	1 (4.5%)	
Unknown	19 (33.3%)	5 (22.7%)	
Rearrangement			
Single hit	27 (47.4%)	3 (13.6%)	< 0.001
Double/triple hit	18 (31.6%)	18 (81.8%)	
Missing *BCL2/BCL6*	12 (21.1%)	1 (4.5%)	
Duration of follow-up (months)			
Mean (SD)	42.9 (24.6)	41.1 (25.8)	0.74
Median [min, max]	39.7 [3.42, 89.6]	41.8 [6.04, 93.3]

**Table 4:** Baseline characteristics of stage II DH/TH DLBCL patients by treatment.

	R-CHOP	More intensive chemotherapy	*p*-value
	(*N* = 18)	(*N* = 18)	
Age (years)			
Mean (SD)	65.9 (11.3)	65.0 (11.2)	0.96
Median [Min, Max]	64.0 [47.0, 84.0]	67.5 [35.0, 82.0]	
Sex			
Male	11 (61.1%)	9 (50.0%)	0.74
Female	7 (38.9%)	9 (50.0%)	
LDH level			
Within reference range	10 (55.6%)	5 (27.8%)	0.18
Elevated	8 (44.4%)	13 (72.2%)	
Extranodal localizations (no.)			
None	15 (83.3%)	11 (61.1%)	0.26
1	3 (16.7%)	7 (38.9%)	
2 or more	0 (0%)	0 (0%)	
IPI-score			
Low	10 (55.6%)	4 (22.2%)	0.1
Low-intermediate	2 (11.1%)	8 (44.4%)	
High-intermediate	1 (5.6%)	1 (5.6%)	
Unknown	5 (27.8%)	5 (27.8%)	
Duration of follow-up (months)			
Mean (SD)	40.7 (23.1)	38.3 (27.5)	0.61
Median [Min, Max]	41.3 [5.03, 86.9]	31.7 [6.04, 93.3]	

## Discussion

In this large population-based study, we demonstrated that a *MYC*-R is a negative prognostic factor for survival outcomes in stage II, but not in stage I DLBCL.

While a rearrangement of the *MYC* oncogene has been widely recognized as a negative prognostic factor in advanced-stage (III–IV) DLBCL, its impact in limited-stage (I–II) DLBCL remains unclear. Previous studies evaluating the impact of MYC on survival include a limited number of patients and did not distinguish stage I and stage II disease, or focused mainly on DH patients [13, 14]. In this study, separate analyses were performed for stage I and stage II patients. This approach allowed us to dissect the relevance of a *MYC*-R and its clinical consequences for each disease stage.

Our results demonstrate that stage I *MYC*-R patients, including those with DH/TH disease, have an excellent survival outcome comparable to patients without a *MYC* rearrangement, which is in line with a previous report involving limited-stage DH DLBCL [14]. Besides, the excellent outcome of stage I *MYC*-R patients in our cohort is similar to outcomes of limited-stage DLBCL patients treated with R-CHOP(-like) regimens as described in earlier cohorts [18, 19]. This challenges the necessity to perform *MYC*-R screening by FISH in patients with stage I DLBCL disease. This should be carefully streamlined on a per-hospital basis since the Ann Arbor stage is not always known prior to biopsy. In stage II (and beyond), screening for a *MYC*-R is inevitable due to its prognostic significance.

The excellent outcome of stage I *MYC*-R DLBCL patients in this study does not justify treatment with more intensive immunochemotherapy regimens. Instead, we propose the inclusion of stage I MYC-R DLBCL patients in potential future studies aiming to reduce treatment intensity.

In contrast, in stage II DLBCL, we found inferior survival outcomes in *MYC*-R patients. Multivariable analyses showed that SH and DH/TH both negatively affected mortality risk and relapse risk. However, due to the limited number of patients in the SH and DH/TH subgroups, no definitive conclusions can be made regarding survival differences between these groups.

The treatment landscape of DH/TH has evolved over time, resulting in more intensive chemotherapeutic regimens available [10].

In retrospective studies on *MYC*-R patients, intensification of immunochemotherapy treatment with regimens such as DA-EPOCH-R or R-CODOX-M/R-IVAC increased PFS [20] and OS, regardless of disease stage [21]. In a recent French retrospective cohort among patients with advanced-stage DH/TH lymphoma, intensive chemotherapy increased PFS, but not OS [22]. In a small subgroup of stage II *MYC*-R patients in our cohort, treatment with more intensive chemotherapeutic regimens did not seem to improve OS or PFS. Despite small patient numbers, this suggests that new treatment strategies for stage II *MYC*-R patients are needed.

The main strength of this study is the use of a large nationwide population-based cancer registry. In the absence of prospective randomized trials in limited-stage DLBCL patients, national registries offer the best data for population-based analysis of treatment outcomes. Our cohort represents the general DLBLC population, as older age, male gender, and characteristics of high disease burden (elevated LDH, involvement of ≥1 extranodal site, WHO performance score 2–4) were associated with a higher mortality or relapse risk in limited-stage DLBCL, as expected [23–26].

Besides, a nationwide, diagnostic screening assessment of the *MYC* rearrangement using FISH was implemented in the Netherlands [27], resulting in access to comprehensive clinical data on *MYC* status. Due to the increased recognition of *MYC* as a potential prognostic factor, the proportion of FISH analyses has increased over the years in stage I and II DLBCL from 18% in 2014 to 83% in 2020. Nevertheless, 1792 patients had to be excluded due to missing *MYC* status. Given the better prognosis of limited-stage disease compared to advanced-stage disease, FISH testing may be less frequently performed in limited-stage patients. We cannot rule out the possibility that excluding patients with missing *MYC* status has introduced some selection bias. Another limitation of this study is that the diagnostic criteria employed align with the revised 4^th^ edition of the WHO classification [17]. Therefore, patients with *MYC* and *BCL6* rearrangements were classified as DH HGBL, instead of the DLBCL-NOS subtype.

The considerable amount of missing WHO performance scores could have caused bias in the interpretation of the multivariable Cox regression analyses. However, the frequency of missing values was similar between *MYC*-R patients and patients without a *MYC* rearrangement in both stages. The lack of available data on toxicity and the cause of death prevents us from conclusively attributing survival differences in stage II *MYC*-rearranged patients vs. patients without a *MYC* rearrangement to either deficient treatment efficacy or an increased level of toxicity. Despite these limitations, national registries offer the best data for population-based analysis of treatment outcomes.

In conclusion, in stage II DLBCL a *MYC*-R is negatively associated with survival. *MYC* FISH is inevitable and should be used as guidance for new treatment strategies for these patients. In stage I DLBCL, however, survival outcomes of patients treated with R-CHOP(-like) regimens are excellent, irrespective of *MYC*-R status. This leaves no justification for more intensive treatment and challenges diagnostic *MYC* fluorescent in situ hybridization (FISH) in stage I DLBCL patients.

### Supplementary information

Supplements

## Data Availability

The data that support the findings of this study are available via the Netherlands Comprehensive Cancer Organization (IKNL). These data are not publicly available, and restrictions apply to the availability of the data used for the current study. However, these data are available upon reasonable request and with the permission of IKNL.
