# Supplementary material for: A MYC-rearrangement is a negative prognostic factor in stage II, but not in stage I diffuse large B-cell lymphoma
Source: Blood Cancer J. 2024 Jan 4;14(1):2. doi: 10.1038/s41408-023-00971-y (PMC10766972; doi:10.1038/s41408-023-00971-y)
Supplement: Supplementary file 1 — Supplements [file 41408_2023_971_MOESM1_ESM.docx]

**A *MYC*-rearrangement is a negative prognostic factor in stage II, but not in stage I diffuse large B-cell lymphoma**

A.V. de Jonge^1,2*^, J.A.A. Bult^3*^, D.F.E. Karssing^1^, M. Nijland^3^, M.E.D. Chamuleau^1,2^, M. Brink^4^

1 Department of Hematology, Amsterdam UMC Location Vrije Universiteit; Amsterdam, The Netherlands

2 Cancer Center Amsterdam, Cancer Biology and Immunology; Amsterdam, The Netherlands

3 Department of Hematology, University Medical Center Groningen, Groningen, the Netherlands.
4 Department of Research and Development, Netherlands Comprehensive Cancer Organization (IKNL), Utrecht, the Netherlands.

* AVDJ and JAAB contributed equally to this study

**SUPPLEMENTARY DATA**

Supplementary Figures 1-2

Supplementary Tables 1-6

**
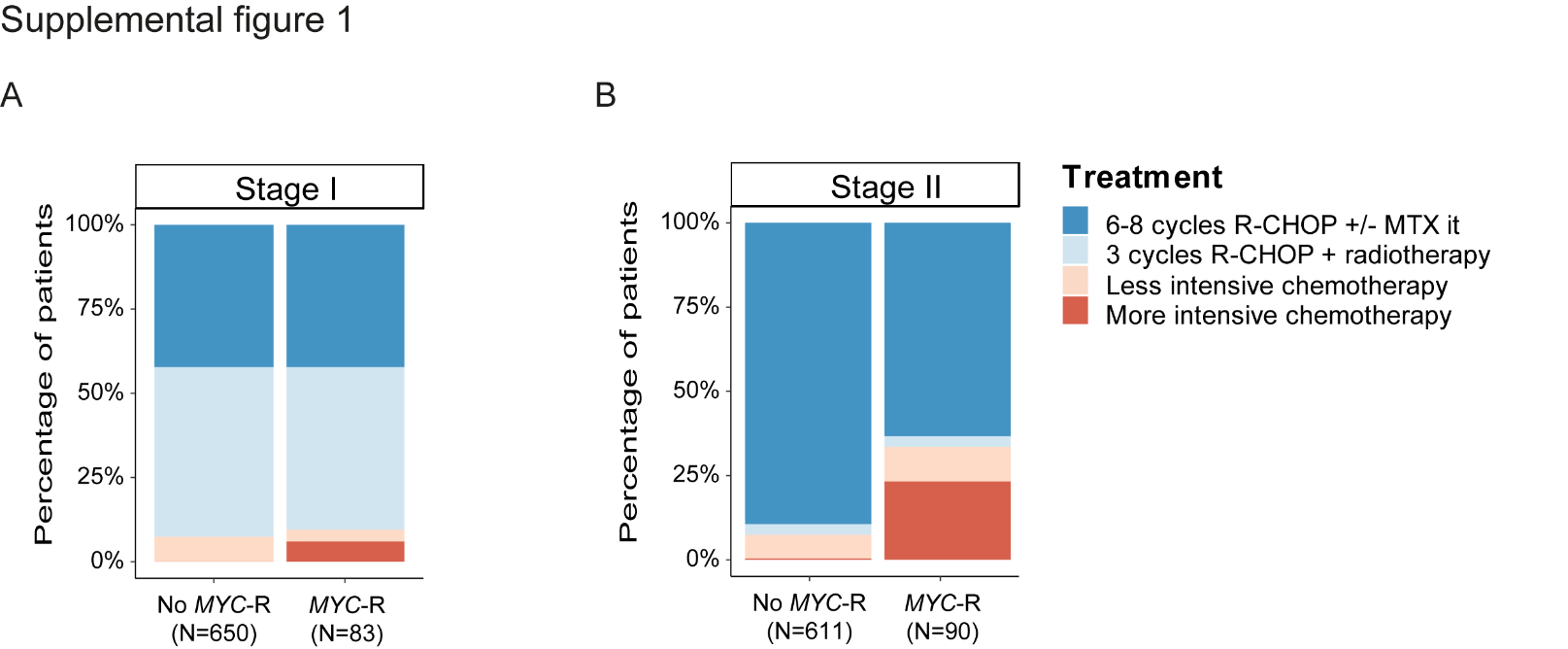
**

**Supplementary Figure 1. Treatment strategies applied in the limited stage DLBCL cohort.**

A-B) Treatment strategies applied in stage I (A) and stage II (B) DLBCL patients without (left column) or with (right column) a *MYC* rearrangement (MYC-R). Data are presented as percentage patients treated with 6-8 cycles R-CHOP +/- methotrexate intrathecal (MTX it, dark blue), 3 cycles of R-CHOP + radiotherapy (light blue), less (light orange) or more (dark orange) intensive chemotherapy of total number of patients.


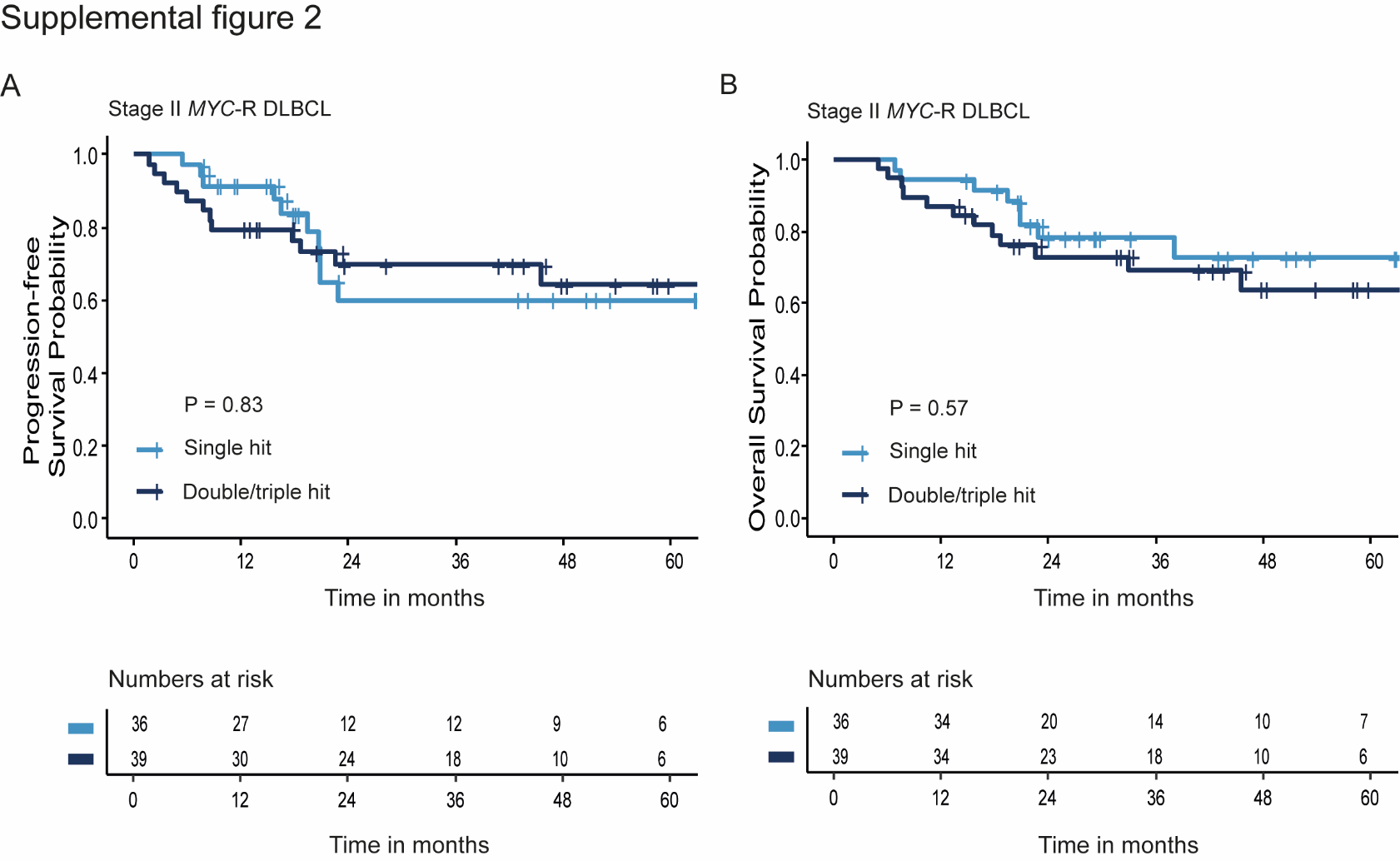


**Supplementary Figure 2. Survival analysis in stage II *MYC*-R DLBCL patients stratified rearrangement status.**

A-B) Progression-free survival (A) and overall survival (B) analyses in stage II *MYC* rearranged (*MYC*-R) DLBCL patients with a single *MYC*-R (single hit, light blue, n=36) or concurrent *BCL2* and/or *BCL6* rearrangements (double/triple hit, dark blue, n=39) using Kaplan-Meier survival analysis.

**Supplemental Table 1.** Limited stage (I-II) DLBCL patients with known *MYC*-R status excluded from analyses based on treatment received.

|  | **Excluded (no, %)**  N=184 |
| --- | --- |
| MBVP treatment  (methotrexate, carmustine, teniposide, prednisone) | 22 (12%) |
| No treatment | 61 (33.2%) |
| Surgery or radiotherapy only | 38 (20.7%) |
| Other treatment | 50 (27.2%) |
| Unclear intention to treat group | 11 (6%) |
| Loss of follow up due to migration to another country | 2 (1.1%) |

**Supplemental Table 2.**

| **Stage I DLBCL** |  |  |
| --- | --- | --- |
|  | **No MYC-R** | **MYC-R** |
|  | (N=650) | (N=83) |
| 6-8 R-CHOP +/- MTX it | 275 (42.3%) | 35 (42.2%) |
| 3 cycles of R-CHOP + radiotherapy | 326 (50.2%) | 40 (48.6%) |
| Less intensive chemotherapy  R-miniCHOP  R-miniCHOP + RT  R-miniCHOP + MTX it  R-miniCHOP + RT + MTx it  R-CVP  R-CEOP | 48 (7.4%)  15  12  4  1  1  15 | 3 (3.6%)  2  1 |
| More intensive chemotherapy  DA-EPOCH-R  R-CHOP + lenalidomide  R-CHOP + atezolizumab | 1 (0.2%)  1 | 5 (6.0%)  4  1 |
|  | | |
| **Stage II DLBCL** |  |  |
|  | **No MYC-R** | **MYC-R** |
|  | (N=611) | (N=90) |
| 6-8 R-CHOP +/- MTX it | 543 (88.9%) | 57 (63.3%) |
| 3 cycles of R-CHOP + radiotherapy | 22 (3.6%) | 3 (3.3%) |
| Less intensive chemotherapy  R-miniCHOP  R-miniCHOP + RT  R-miniCHOP + MTX it  R-CVP  R-CEOP | 43 (7.0%)  28  5  1  2  7 | 8 (8.9%)  6  2 |
| More intensive chemotherapy | 3 (0.5%) | 22 (24.4%) |
| R-CHOEP  DA-EPOCH-R  R-CHOP + lenalidomide  R-CHOP + atezolizumab | 1  2 | 2  14  6 |

**Supplemental Table 3.** Multivariable Cox regression analysis for risk of mortality in stage I DLBCL patients

|  | **Univariable** | | | **Multivariable** | | |
| --- | --- | --- | --- | --- | --- | --- |
|  | **HR** | **95% CI** | **P-value** | **HR** | **95% CI** | **P-value** |
| **Sex** |  |  |  |  |  |  |
| Female | 1 | Reference |  | 1 | Reference |  |
| Male | 1.28 | 0.80 – 2.06 | 0.31 | 1.72 | 1.06 – 2.80 | 0.03 |
| **Age (continuous)** | 1.09 | 1.07 - 1.12 | <0.01 | 1.10 | 1.07 – 1.13 | <0.01 |
| **WHO performance score** |  |  |  |  |  |  |
| <2 | 1 | Reference |  | 1 | Reference |  |
| ≥2 | 4.60 | 2.23 - 9.53 | <0.01 | 2.92 | 1.37 – 6.22 | 0.01 |
| Unknown | 0.88 | 0.54 - 1.43 | 0.60 | 0.79 | 0.47 – 1.33 | 0.37 |
| **Number of extranodal sites** |  |  |  |  |  |  |
| <1 | 1 | Reference |  |  |  |  |
| ≥1 | 1.31 | 0.84 - 2.04 | 0.24 |  |  |  |
| Unknown | 0.00 | 0.00 - inf | 1.00 |  |  |  |
| **LDH level** |  |  |  |  |  |  |
| Within reference range | 1 | Reference |  | 1 | Reference |  |
| Elevated | 2.61 | 1.62 - 4.18 | <0.01 | 2.25 | 1.38 – 3.68 | <0.01 |
| Unknown | 2.06 | 0.81 - 5.19 | 0.13 | 1.94 | 0.74 – 5.08 | 0.18 |
| **Rearrangement** |  |  |  |  |  |  |
| No *MYC-*R | 1 | Reference |  | 1 | Reference |  |
| Single hit | 0.29 | 0.04 - 2.06 | 0.22 | 0.28 | 0.04 – 2.09 | 0.21 |
| Double or triple hit | 0.70 | 0.22 - 2.22 | 0.54 | 0.58 | 0.18 – 1.88 | 0.37 |
| Missing *BCL2/BCL6* | 0.81 | 0.20 - 3.29 | 0.77 | 0.37 | 0.09 – 1.61 | 0.19 |
| **Treatment group** |  |  |  |  |  |  |
| 6-8 R-CHOP +/- MTX it | 1 | Reference |  |  |  |  |
| 3 cycles of R-CHOP  + radiotherapy | 0.87 | 0.54 - 1.40 | 0.58 |  |  |  |
| Less intensive chemotherapy | 3.09 | 1.61 - 5.91 | <0.01 |  |  |  |
| More intensive chemotherapy | 0.00 | 0.00 - Inf | 1.00 |  |  |  |

**Supplemental Table 4.** Multivariable Cox regression analysis for risk of relapse in stage I DLBCL patients

|  | **Univariable** | | | **Multivariable** | | |
| --- | --- | --- | --- | --- | --- | --- |
|  | **HR** | **95% CI** | **P-value** | **HR** | **95% CI** | **P-value** |
| **Sex** |  |  |  |  |  |  |
| Female | 1 | Reference |  | 1 | Reference |  |
| Male | 1.41 | 0.92 – 2.16 | 0.12 | 1.69 | 1.09 – 2.63 | 0.02 |
| **Age (continuous)** | 1.06 | 1.04 - 1.09 | <0.01 | 1.06 | 1.04 – 1.09 | <0.01 |
| **WHO performance score** |  |  |  |  |  |  |
| <2 | 1 | Reference |  | 1 | Reference |  |
| ≥2 | 3.86 | 1.96 - 7.61 | <0.01 | 2.53 | 1.23 – 5.21 | 0.01 |
| Unknown | 0.92 | 0.58 - 1.39 | 0.62 | 0.91 | 0.57 – 1.44 | 0.68 |
| **Number of extranodal sites** |  |  |  |  |  |  |
| <1 | 1 | Reference |  |  |  |  |
| ≥1 | 1.15 | 0.78 - 1.71 | 0.48 |  |  |  |
| Unknown | 0.00 | 0.00 - inf | 1.00 |  |  |  |
| **LDH level** |  |  |  |  |  |  |
| Within reference range | 1 | Reference |  | 1 | Reference |  |
| Elevated | 2.88 | 1.90 - 4.36 | <0.01 | 2.28 | 1.47 – 3.55 | <0.01 |
| Unknown | 1.67 | 0.67 - 4.18 | 0.28 | 1.77 | 0.69 – 4.54 | 0.24 |
| **Rearrangement** |  |  |  |  |  |  |
| No *MYC-*R | 1 | Reference |  | 1 | Reference |  |
| Single hit | 0.25 | 0.03 - 1.79 | 0.17 | 0.30 | 0.04 – 2.19 | 0.23 |
| Double or triple hit | 1.90 | 0.96 - 3.78 | 0.07 | 1.58 | 0.75 – 3.34 | 0.23 |
| Missing *BCL2/BCL6* | 1.06 | 0.34 - 3.37 | 0.92 | 0.69 | 0.21 – 2.24 | 0.54 |
| **Treatment group** |  |  |  |  |  |  |
| 6-8 R-CHOP +/- MTX it | 1 | Reference |  | 1 | Reference |  |
| 3 cycles of R-CHOP  + radiotherapy | 0.64 | 0.42 - 0.98 | 0.05 | 0.63 | 0.41 – 0.98 | 0.04 |
| Less intensive chemotherapy | 2.20 | 1.21 - 4.02 | 0.01 | 1.31 | 0.67 - 2.55 | 0.42 |
| More intensive chemotherapy | 1.68 | 0.23 - 12.25 | 0.61 | 1.69 | 0.21 – 13.96 | 0.63 |

**Supplemental Table 5.** Multivariable Cox regression analysis for risk of mortality in stage II DLBCL patients

|  | **Univariable** | | | **Multivariable** | | |
| --- | --- | --- | --- | --- | --- | --- |
|  | **HR** | **95% CI** | **P-value** | **HR** | **95% CI** | **P-value** |
| **Sex** |  |  |  |  |  |  |
| Female | 1 | Reference |  |  |  |  |
| Male | 1.02 | 0.67 – 1.54 | 0.93 |  |  |  |
| **Age (continuous)** | 1.08 | 1.06 – 1.10 | <0.01 | 1.08 | 1.06 – 1.11 | <0.01 |
| **WHO performance score** |  |  |  |  |  |  |
| <2 | 1 | Reference |  |  |  |  |
| ≥2 | 1.73 | 0.69 - 4.35 | 0.24 |  |  |  |
| Unknown | 1.07 | 0.70 - 1.65 | 0.75 |  |  |  |
| **Number of extranodal sites** |  |  |  |  |  |  |
| <1 | 1 | Reference |  | 1 | Reference |  |
| ≥1 | 1.40 | 0.93 - 2.12 | 0.11 | 1.63 | 1.07 – 2.49 | 0.02 |
| **LDH level** |  |  |  |  |  |  |
| Normal | 1 | Reference |  |  |  |  |
| Elevated | 0.97 | 0.63 - 1.49 | 0.89 |  |  |  |
| Unknown | 1.46 | 0.20 - 10.54 | 0.71 |  |  |  |
| **Rearrangement** |  |  |  |  |  |  |
| No *MYC-*R | 1 | Reference |  | 1 | Reference |  |
| Single hit | 2.59 | 1.29 - 5.20 | 0.01 | 2.27 | 1.12 – 4.59 | 0.02 |
| Double or triple hit | 3.18 | 1.72 - 5.87 | <0.01 | 3.55 | 1.90 – 6.65 | <0.01 |
| Missing *BCL2/BCL6* | 0.92 | 0.22 - 3.75 | 0.90 | 1.26 | 0.30 – 5.20 | 0.75 |
| **Treatment group** |  |  |  |  |  |  |
| 6-8 R-CHOP +/- MTX it | 1 | Reference |  |  |  |  |
| 3 cycles of R-CHOP  + radiotherapy | 2.30 | 0.92 - 5.72 | 0.08 |  |  |  |
| Less intensive chemotherapy | 4.31 | 2.48 - 7.48 | <0.01 |  |  |  |
| More intensive chemotherapy | 2.09 | 0.84 - 5.18 | 0.11 |  |  |  |

**Supplemental Table 6.** Multivariable Cox regression analysis for risk of relapse in stage II DLBCL patients

|  | **Univariable** | | | **Multivariable** | | |
| --- | --- | --- | --- | --- | --- | --- |
|  | **HR** | **95% CI** | **P-value** | **HR** | **95% CI** | **P-value** |
| **Sex** |  |  |  |  |  |  |
| Female | 1 | Reference |  |  |  |  |
| Male | 1.23 | 0.85 - 1.77 | 0.27 |  |  |  |
| **Age (continuous)** | 1.05 | 1.03 – 1.07 | <0.01 | 1.05 | 1.03 – 1.06 | <0.01 |
| **WHO performance score** |  |  |  |  |  |  |
| <2 | 1 | Reference |  |  |  |  |
| ≥2 | 1.22 | 0.49 - 3.03 | 0.66 |  |  |  |
| Unknown | 1.06 | 0.73 - 1.54 | 0.75 |  |  |  |
| **Number of extranodal sites** |  |  |  |  |  |  |
| <1 | 1 | Reference |  |  |  |  |
| ≥1 | 1.13 | 0.79 - 1.61 | 0.5 |  |  |  |
| **LDH level** |  |  |  |  |  |  |
| Normal | 1 | Reference |  |  |  |  |
| Elevated | 0.98 | 0.67 - 1.42 | 0.92 |  |  |  |
| Unknown | 1.14 | 0.16 - 8.18 | 0.90 |  |  |  |
| **Rearrangement** |  |  |  |  |  |  |
| No *MYC-*R | 1 | Reference |  | 1 | Reference |  |
| Single hit | 2.37 | 1.27 - 4.43 | 0.01 | 2.20 | 1.17 – 4.14 | 0.01 |
| Double or triple hit | 2.19 | 1.2 - 3.99 | 0.01 | 2.08 | 1.14 – 3.79 | 0.02 |
| Missing *BCL2/BCL6* | 1.03 | 0.33 - 3.27 | 0.96 | 1.30 | 0.41 – 4.13 | 0.65 |
| **Treatment group** |  |  |  |  |  |  |
| 6-8 R-CHOP +/- MTX it | 1 | Reference |  |  |  |  |
| 3 cycles of R-CHOP  + radiotherapy | 1.88 | 0.82 - 4.3 | 0.13 |  |  |  |
| Less intensive chemotherapy | 3.09 | 1.84 - 5.2 | <0.01 |  |  |  |
| More intensive chemotherapy | 1.38 | 0.56 - 3.39 | 0.49 |  |  |  |
